# dot-app: a Graphviz-Cytoscape conversion plug-in

**DOI:** 10.12688/f1000research.9751.2

**Published:** 2017-07-10

**Authors:** Braxton Fitts, Ziran Zhang, Massoud Maher, Barry Demchak

**Affiliations:** 1 Department of Computer Science and Engineering, UC San Diego, San Diego, USA; 2 Department of Health Sciences, UC San Diego, San Diego, USA

**Keywords:** Network, import, export, format conversion, attribute conversion, data visualization, Cytoscape, GraphViz, DOT

## Abstract

dot-app is a Cytoscape 3 app that allows Cytoscape to import and export Graphviz (.dot, .gv) files, also known as DOT files due to the .dot extension and their conformance to the DOT language syntax. The DOT format was originally created in the early 2000s to represent graph topologies, layouts and formatting. DOT-encoded files are produced and consumed by a number of open-source graph applications, including Graphviz, Gephi, Tulip, and others. While DOT-based graph applications are popular, they emphasize general graph layout and styling over the topological and semantic analysis functions available in domain-focused applications such as Cytoscape. While domain-focused applications have easy access to large networks (10,000 to 100,000 nodes) and advanced analysis and formatting, they do not have as many styling options as the Graphviz software suite. dot-app enables the interchange of networks between Cytoscape and DOT-compatible applications so that users can benefit from the features of both. dot-app was first deployed to the Cytoscape App Store in August 2015, has since registered more than 1,200 downloads, and has been highly rated by more than 20 users.

## Introduction

Cytoscape ^1^ is a popular tool for visualizing and analyzing networks used in scientific and commercial analysis, most commonly in bioinformatics. It enables users to discover and load curated and uncurated networks representing molecular and genomic interactions, load ad-hoc or custom networks, and share networks that others have created. Once networks are loaded, users can manually annotate a network or automatically integrate annotations using a number of algorithms and databases. Users can perform a number of graph-oriented and semantic-aware analyses ranging from graph statistics to motif and cluster discovery to upstream and downstream structural and functional inferences. Users can also perform a number of complex graph filtering and layout operations to drive and focus the semantic understanding of network interactions and structure.

Even beyond analysis and layout, users commonly derive and demonstrate network meaning by using visual cues to distinguish relationships and attributes. For this, Cytoscape provides a visual style system that enables users to paint nodes and edges using color, border thickness, size, fonts, arrows, and other devices.

Much of the power and functionality of Cytoscape is delivered as apps available in the Cytoscape App Store ( http://apps.cytoscape.org). The store contains 319 apps (June 2017) that provide a range of functionality from file import/export to analysis to visualization and publishing. Based on the success of the combination of Cytoscape core and downloadable apps, Cytoscape is downloaded approximately 14,000 times per month worldwide and is started approximately 3,000 times each working day. As of 2015, Cytoscape has been cited in 700 academic peer-reviewed papers per year.

While Cytoscape is the dominant network analysis and visualization platform in bioinformatics, it is not the only platform. To support interoperability with a number of network-oriented workflows and applications, Cytoscape offers a number of natively supported file import/export modules, and leverages a number of them that are available as apps in the App Store. Some of these file formats ^2^ do not include visual information, such as SIF (.sif) and NNF (.nnf). Other file formats, such as GEXF (.gexf) and XGMML(.xgmml), include varying amounts of visual information. Our primary motivation for creating dot-app was to extend Cytoscape’s interoperability to users that primarily use Graphviz software. Prior to dot-app, a user would either have to convert the network to a file format compatible with Cytoscape or attempt to manually reconstruct the network. Converting a DOT file into a Cytoscape network by use of an intermediary file format is an inherently bad process because each conversion results in a loss of some data. Furthermore, reconstructing a graph manually in Cytoscape might not be too difficult for a simple graph with few nodes and edges, but reconstruction would become more time consuming for larger networks which have nodes of distinct visual properties since each node would have to be created one at a time and these visual properties have to be set separately for each node if there is no underlying data to which the visual properties can be mapped. With dot-app, we minimize information loss by providing a direct conversion from DOT to a Cytoscape network and we reduce the time needed to reconstruct the DOT network in Cytoscape.

Graphviz ^3^ is a popular, well-established graph visualization software suite that produces DOT (.dot, .gv) files containing graph structure, layout, and styling information; and uses these files to output these networks in a variety of file formats. These files adhere to the DOT language syntax ( http://www.graphviz.org/doc/info/lang.html). In this paper, we use “DOT file/network” and “Graphviz file/network” interchangeably. Due to Graphviz’s continued usage, many graph visualization and analysis applications support the import and export of DOT files. Table 1 lists some of these applications and whether they can import or export DOT files.

**Table 1.** Some graph visualization and analysis software and their compatibility with DOT files.

Graph Software	Import DOT files	Export DOT files
Tulip	Supported	Not Supported
Gephi	Supported	Not Supported
NetworkX	Supported	Supported
SocNetV	Supported	Not Supported

**Figure 1.**
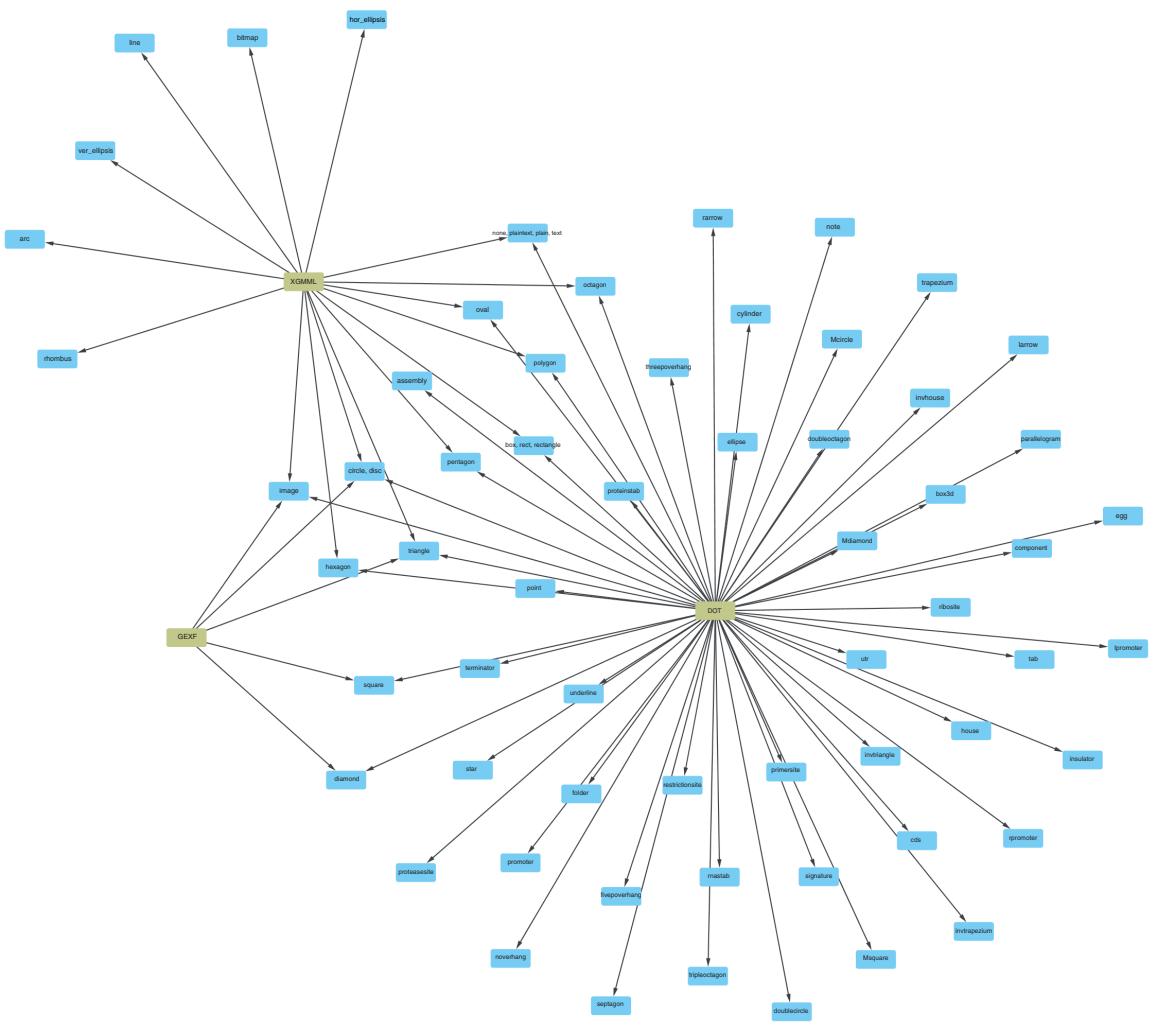
Graph showing which node shapes are supported by which file format.

As mentioned earlier, Cytoscape provides support for XGMML ( http://www.cs.rpi.edu/research/groups/pb/punin/public_html/XGMML/) and GEXF ( http://www.gexf.net/format/index.html), which are two file formats that support visual information such as node shapes and line types. In comparison to GEXF and XGMML, the DOT format defines more node shapes, arrow shapes, and has unique features such as gradients for node colors and edges painted with multiple colors. Table 2 shows some options for edge line types and which file formats support them. GEXF’s entry for multiple lines is marked with an asterisk because its support for the line type is limited. Unlike DOT which supports any number of lines for an edge, GEXF only supports up to two lines for an edge with its “double” line type. Figure 1 is a graph of all the node shapes between the three formats and shows which formats support which node shapes. Names for the same node shape share a node in the graph, such as the “disc” shape name found in GEXF and the “circle” shape name found in both DOT and XGMML.

**Table 2.** Edge line types available with DOT, XGMML, or GEXF. X’s are supported types.

Line Types/ File Format	Solid	Dotted	Dashed	Tapered	Multiple
**DOT**	**X**	**X**	**X**	**X**	**X**
**GEXF**	**X**	**X**	**X**		X*
**XGMML**	**X**		**X**		

DOT files contain a number of visual attributes that map well to Cytoscape visualization functionality, and vice versa. However, incompatibilities do exist where some Cytoscape features cannot be represented in DOT, or where DOT represents some features that cannot be realized in Cytoscape. These incompatibilities are described in "Conversion details" section.

We present dot-app as a Cytoscape app that implements both the import and export of graphs encoded in DOT files. We describe the operation of dot-app, how dot-app maps Cytoscape networks to DOT networks and vice versa, issues that arise because of incompatibilities between the Cytoscape and DOT network models, representative use cases, and prospects for future work.

## Operation

### Minimum system requirement

Dot-app requires Java 7 or above and Cytoscape v3.2 or above.

### Installation

Open Cytoscape and navigate to the App Manager from the menu (“Apps->App Manager”). While in the “Install Apps” tab, type dot-app in the search bar. Make sure the download site is http://apps.cytoscape.org, as our app is in the Cytoscape App Store. Click on dot-app to select it and then click the install button to download and install the app.

### Import

A Graphviz network can be imported in three ways: from the welcome screen (via the “From Network File…” button), from the menu (“File->Import->Network->File…”), or from the toolbar (by clicking the “Import Network from File” button).

**Figure 2.**
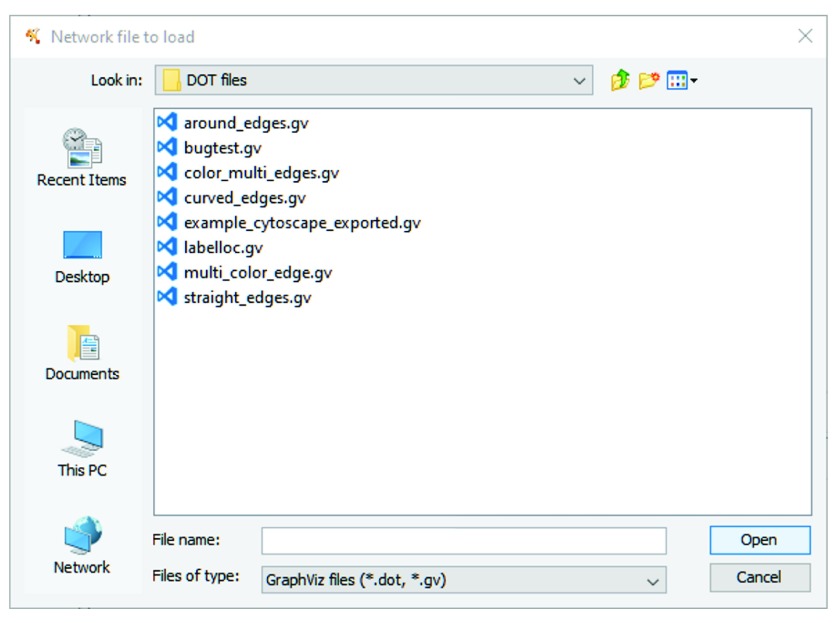
Network file to load dialog with Graphviz files selected.

Users are presented with a file browser dialog titled “Network file to load” (as in Figure 2). The user is able to filter the dialog to display only Graphviz files by selecting “GraphViz files (*.gv, *.dot)” from the drop-down menu for “Files of Type”. Note that no difference exists between a Graphviz file with an extension of .dot and a Graphviz file with an extension of .gv. However, the .gv extension is preferred because versions of Microsoft Word also use the .dot extension ( https://marc.info/?l=graphviz-devel&m=129418103126092). From this point, importing a Graphviz network is the same as importing a network from any of Cytoscape's accepted file formats. Those steps are detailed in the Cytoscape User Manual ( http://manual.cytoscape.org/en/stable/).

### Export

To export a Cytoscape network as a Graphviz network, use “Export -> Network and View” from the menu. (Using "Export -> Network" is also possible, but this will result in a Graphviz file that contains no visual information and a notification to use "Export -> Network and View" instead.)

**Figure 3.**
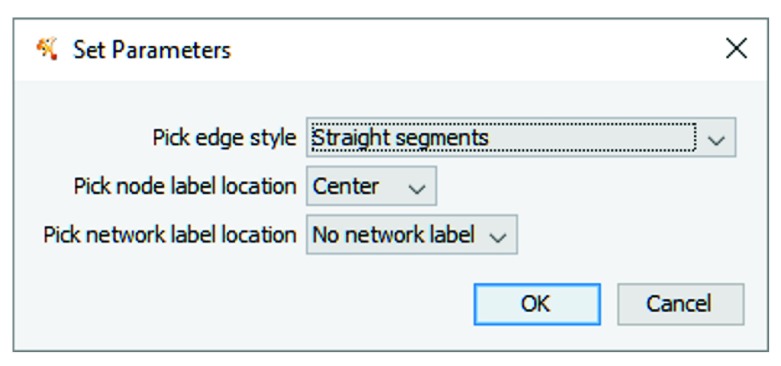
Set Parameter pop-up prompts users with three options.

Selecting “GraphViz files (*.dot,*.gv)” in the Export dialog launches dot-app and prompts the user to choose from three options, as shown in Figure 3 below. The purposes of these options are explained in the following section.

### Set Parameters prompt

**Figure 4.**
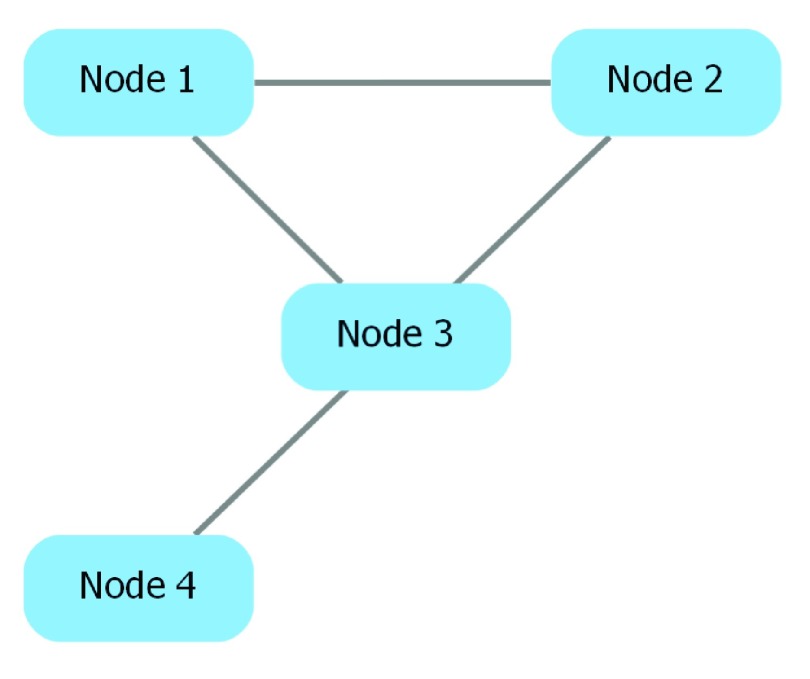
The network exported with the “Straight segments” option, as “splines = ‘false’” in the output Graphviz file.

**Figure 5.**
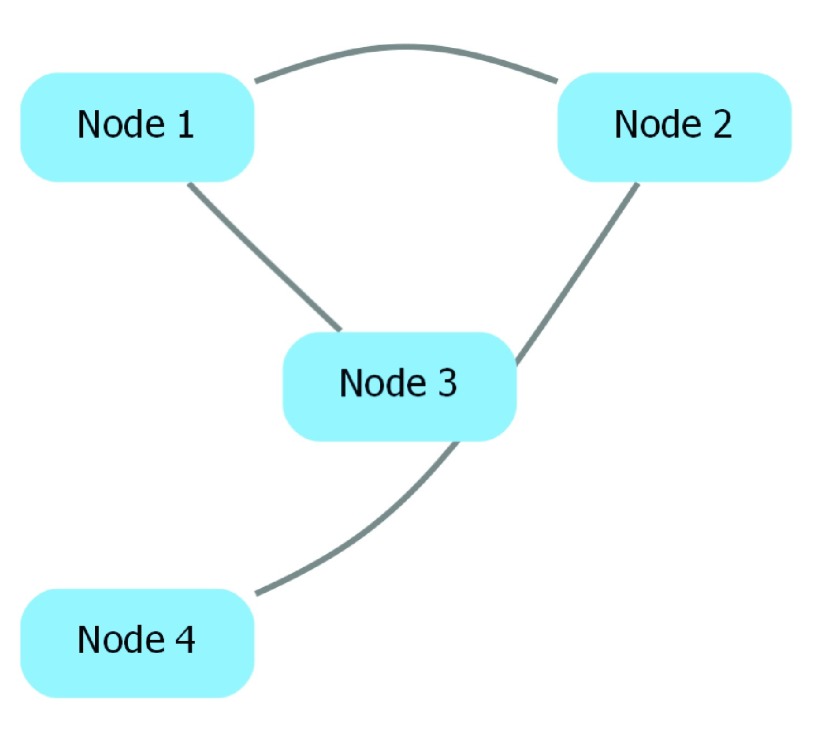
The network exported with the “Curved segments” option, as “splines = ‘curved’” in the output Graphviz file.

**Figure 6.**
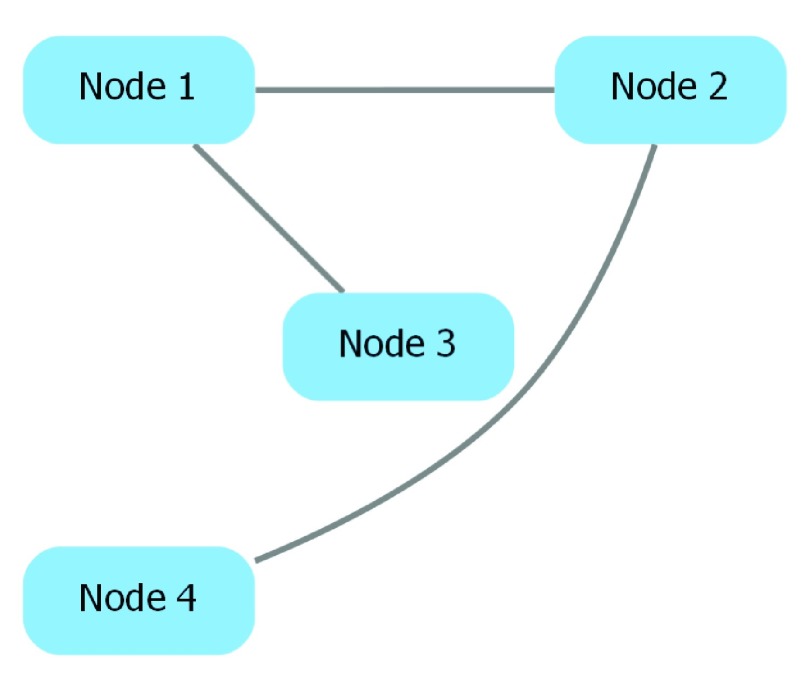
The network exported with the “Curved segments routed around nodes” option, as “splines = ‘true’” in the output Graphviz file.

***Pick edge style***. Cytoscape provides edge-routing capabilities that cannot be conserved during the export process, so dot-app provides three edge routing options: “Straight segments”, “Curved segments”, and “Curved segments routed around nodes”. These options change the value of the “splines” attribute that appears in the exported Graphviz file. The Graphviz file for a network exported from Cytoscape is shown below, and the attribute modified by the “Pick Edge Style” option is underlined and in bold. Figure 4, Figure 5, and Figure 6 depict pictures of the network with each edge style chosen.


graph example {
bgcolor = "#FFFFFFFF"

                        **
                            splines = "false"
                        **
outputorder = "edgesfirst"
esep = "0"
pad = "2"
node [label = "",penwidth = "0.000000",height = "0.486111",width = "1.041667",tooltip = "",color = "#CCCCCCFF",fillcolor = "#89D0F5FF",shape = "rectangle",style = "solid,rounded,filled",fontname = "SansSerif.plain",fontsize = "12",fontcolor = "#000000FF",fixedsize = "true",labelloc = "c"]
edge [label = "",penwidth = "2.000000",tooltip = "",arrowhead = "none",arrowtail = "none",color = "#848484FF",fontname = "Dialog.plain",fontsize = "10",fontcolor = "#000000FF",style = "solid",dir = "both"]
"Node 1§64" [label = "Node 1",pos = "-243.000000,42.000000"]
"Node 2§66" [label = "Node 2",pos = "-70.975037,42.014648"]
"Node 3§68" [label = "Node 3",pos = "-159.020569,-41.005199"]
"Node 4§70" [label = "Node 4",pos = "-243.001038,-123.001381"]
"Node 1§64" -- "Node 2§66"
"Node 2§66" -- "Node 4§70"
"Node 1§64" -- "Node 3§68"
}



**Figure 7.**
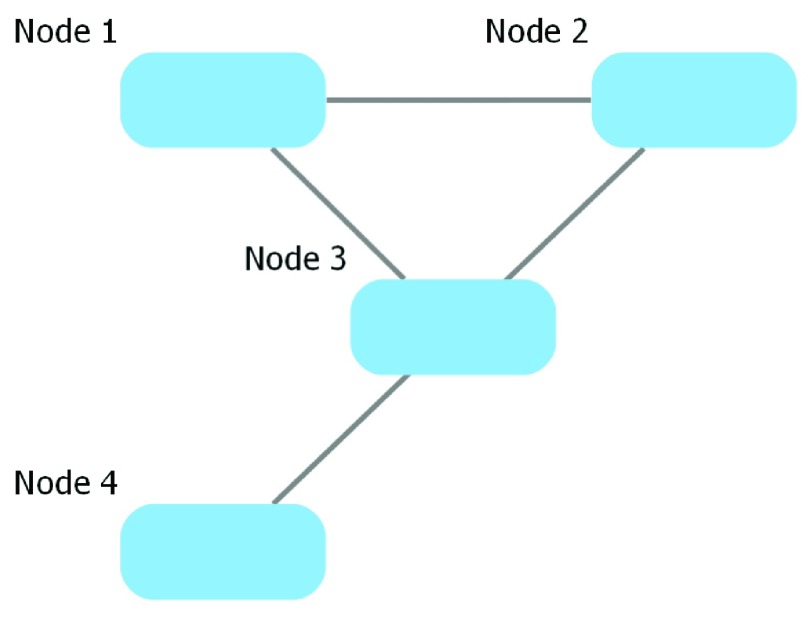
A network exported with the “External” option for node label location.

***Pick node label location***. Graphviz does not offer the flexible label placement that Cytoscape offers. As such, dot-app gives the options of “Center,” “Top,” “Bottom,” and “External” to allow the user to specify the label location applied to every node. In the output Graphviz file, the “Center”, “Top”, and “Bottom” options change the value of the “labelloc” attribute that appears in the node default attribute list. The options respectively change the value to “c”, “t”, and “b”. In contrast, the “External” option causes the node labels to set the “xlabel” attribute instead of the “label” attribute in the output Graphviz file. The “xlabel” attribute causes the label to be placed in a location near its node that does not cause it to overlap with any other nodes or labels. Figure 7 shows a network exported with the “External” option.

***Pick network label location***. dot-app provides the options “No network label,” “Top,” and “Bottom” to allow the user to specify whether the network itself should be labeled and, if so, where the label is placed. The options “Top” and “Bottom” cause the “labelloc” attribute and “label” attribute for the graph to be written to the output Graphviz file. Furthermore, the “label” attribute will be set to the network’s name in Cytoscape. In contrast, the “No network label” option omits both the “labelloc” attribute and the “label” attribute.

## Implementation

### Import

**Figure 8.**
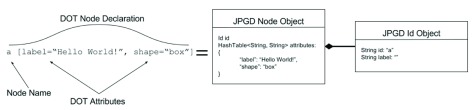
DOT Node Declaration to JPGD Node Object.

For the import function of dot-app, we used Java-based Parser for Graphviz Documents (JPGD), a Graphviz document parser made by Alexander Merz ( http://www.alexander-merz.com/graphviz/). We use JPGD to read the DOT file and create a JPGD data model of the graph or collection of graphs in the file. A JPGD Graph object is created for each graph in the file, a JPGD Node object for each node, and a JPGD Edge object for each edge. Each JPGD object contains the DOT attributes for the graph element they represent. Figure 8 provides a high-level picture of the conversion of a DOT node declaration to the JPGD Node object. Detailed information about the JPGD objects can be found on JPGD’s website ( http://www.alexander-merz.com/graphviz/doc.html).

Once the JPGD data model is built, we begin creation of the network in Cytoscape by using the JPGD data model to create a Cytoscape data model. A CyNetwork object is created for each JPGD Graph object, a CyNode object for each JPGD Node object, and a CyEdge object for each JPGD Edge object. At this point in the importation, the graph elements themselves have been imported, but not their visual attributes.

**Figure 9.**
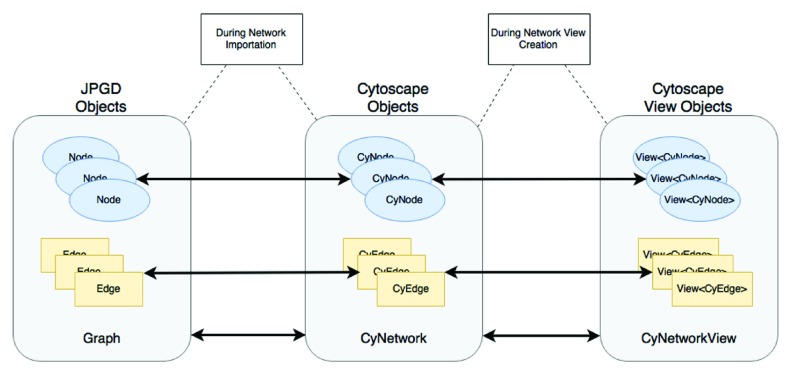
Relationships between JPGD objects, Cytoscape objects and Cytoscape view objects.

A Cytoscape View data model has to be created in order for the DOT graphs to be visually recreated in Cytoscape. In order to facilitate this, we associate each JPGD object to their corresponding Cytoscape object and store these associations in maps. These maps are necessary because the Cytoscape View data objects are created from the Cytoscape objects, yet the visual attributes for each element is stored in the JPGD objects. When the network view is being built in Cytoscape, our Reader objects use these associations to create the Cytoscape View objects. Three Reader classes exist: NetworkReader, NodeReader, and EdgeReader. At the start of the network view creation, a VisualStyle object is created for the network. This VisualStyle is constructed so that if the DOT file does not specify default attributes then the default attributes are already set to DOT implicit values. Each Reader object uses the VisualStyle to set the default attributes for its class of graph components. In addition, each Reader iterates through their association map to retrieve the View objects for the graph components. After the View objects are retrieved, the DOT attributes and their assigned values are converted into their Cytoscape equivalents, and the resulting VisualProperty and VisualPropertyValue are set to the View. If a DOT attribute does not have an equivalent VisualProperty or its assigned value does not have an equivalent VisualPropertyValue then it is ignored. Figure 9 shows the high-level relationships among the JPGD objects, the Cytoscape objects, and the Cytoscape View objects.

### Export

We created three classes—NodePropertyMapper, EdgePropertyMapper and NetworkPropertyMapper—to accomplish the export function of dot-app. Each Mapper class contains an ArrayList into which the Mapper classes insert the DOT attribute strings for easily convertible Cytoscape VisualProperties. In addition, each Mapper class has unique helper methods that create the DOT attribute strings for the DOT attributes that have values determined by multiple Cytoscape VisualProperties. One such attribute is the “style” DOT attribute, the value of which is determined by NODE_SHAPE, NODE_BORDER_LINE_TYPE, and NODE_VISIBLE. The NodePropertyMapper class handles the conversion of the CyNodes and their VisualProperties into their DOT string equivalents. The EdgePropertyMapper class handles the conversion of the CyEdges and their VisualProperties into their DOT string equivalents. Finally, the NetworkPropertyMapper class handles the conversion of the CyNodes' and CyEdges' default VisualProperties and the CyNetwork’s VisualProperties into their DOT string equivalents. In order to build the final DOT attribute declaration string for each element, we concatenate all of the individual DOT attribute strings together.

## Conversion details

### Import

***Supported DOT attributes***. The DOT attributes in the following subsections contribute to the Cytoscape network during the import process. Most of the DOT attributes listed below correspond to a single Cytoscape visual property and their values are able to copied over easily (such as the label attribute), but a few of the attributes either affect multiple visual properties at once (e.g., the “style” DOT attribute, as described below) or a conversion needs to performed on their values (e.g., the “height” and “width” attributes) due to the fact that the information is stored differently between the DOT model and the Cytoscape model. If a DOT attribute is assigned a value that does not have an equivalent value for the corresponding Cytoscape visual property, then the DOT attribute is ignored. The “weight” DOT attribute is imported as an Edge table attribute (i.e., data) because no corresponding Cytoscape visual property exists. The DOT attributes listed in File 1 of the Supplementary Material are ignored and have no effect on the visualization in Cytoscape.

***Node DOT attributes***. Table 3 lists the DOT attributes that can apply to nodes and the specific Cytoscape visual properties to which they map. The “pos” attribute maps to both NODE_X_POSITION and NODE_Y_POSITION because the value of the “pos” attribute is a coordinate pair of the form “x, y”.

**Table 3.** DOT node attributes and their corresponding Cytoscape visual properties.

DOT attribute	Cytoscape visual property
label	NODE_LABEL
xlabel	NODE_LABEL
color	NODE_BORDER_PAINT
fillcolor	NODE_FILL_COLOR
penwidth	NODE_BORDER_WIDTH
width	NODE_WIDTH
height	NODE_HEIGHT
shape	NODE_SHAPE
fontname	NODE_LABEL_FONT_FACE
fontsize	NODE_LABEL_FONT_SIZE
fontcolor	NODE_LABEL_FONT_COLOR
style	See section “The ‘style’ DOT Attribute”
pos	NODE_X_POSITION, NODE_Y_POSITION
tooltip	NODE_TOOLTIP

***Edge DOT attributes***. Table 4 lists the DOT attributes that apply to edges and the specific Cytoscape visual properties to which they map.

**Table 4.** DOT edge attributes and their corresponding Cytoscape visual properties.

DOT attribute	Cytoscape visual property
label	EDGE_LABEL
xlabel	EDGE_ LABEL
weight	No Visual Property
color	EDGE_UNSELECTED_PAINT
penwidth	EDGE_WIDTH
fontname	EDGE_LABEL_FONT_FACE
fontsize	EDGE_LABEL_FONT_SIZE
fontcolor	EDGE_LABEL_FONT_COLOR
style	See section “The ‘style’ DOT Attribute”
arrowhead	EDGE_TARGET_ARROW_SHAPE
arrowtail	EDGE_SOURCE_ARROW_SHAPE
tooltip	EDGE_TOOLTIP

***The “style” DOT attribute***. The “style” DOT attribute applies to both nodes and edges. The attribute takes a comma-separated list of keywords as its value. These keywords directly affect which Cytoscape visual properties are modified. Table 5 lists the keywords that dot-app supports, the graph components they affect and the Cytoscape visual properties to which the keywords map.

**Table 5.** The keywords for the DOT style attribute, the graph element that it can affect, and the Cytoscape visual properties that are modified as a result (the “rounded” keyword only affects NODE_SHAPE if the shape="rectangle").

“Style” attribute keyword	Affects nodes or edges	Cytoscape visual property
solid	Nodes/Edges	NODE_BORDER_LINE_TYPE EDGE_LINE_TYPE
dashed	Nodes/Edges	NODE_BORDER_LINE_TYPE EDGE_LINE_TYPE
dotted	Nodes/Edges	NODE_BORDER_LINE_TYPE EDGE_LINE_TYPE
invis	Nodes/Edges	NODE_VISIBLE EDGE_VISIBLE
rounded	Nodes	NODE_SHAPE*
filled	Nodes	NODE_TRANSPARENCY

***The “weight” DOT attribute***. During the import of a network using dot-app, a weight column is added to the Cytoscape network’s edge table. If the “weight” attribute is supplied for an edge, its value is assigned to the weight column entry for the edge.

***The The “height” and “width” DOT attributes***. In the DOT language, the height and width of nodes are specified in inches. However, the height and width of nodes are specified in points in Cytoscape. During both import and export we handle the necessary unit conversion (1 in = 72 pts) in order to maintain the correctness of the graph.

**Figure 10.**
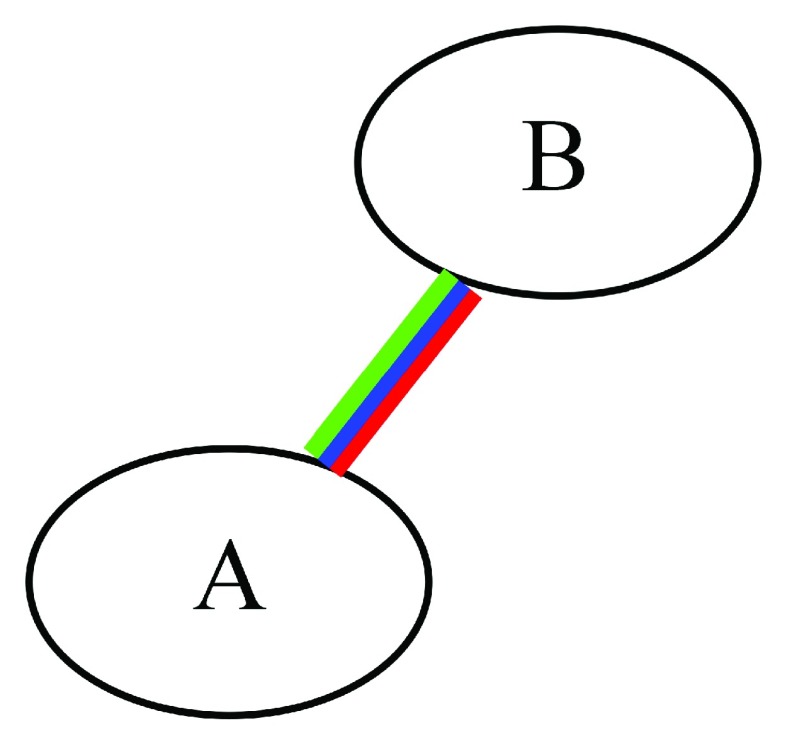
Example of an edge rendered as colored parallel lines.

**Figure 11.**
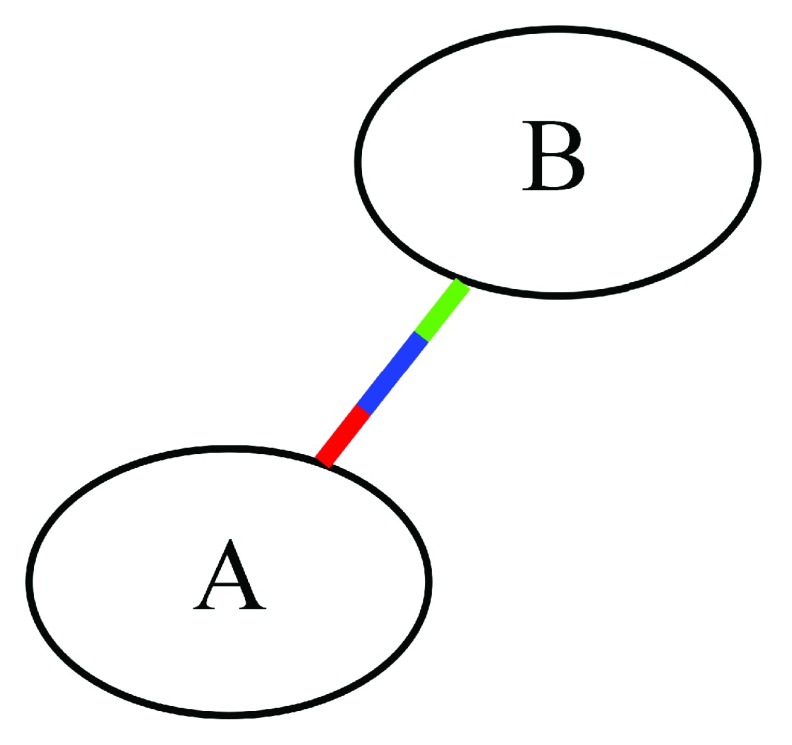
Example of an edge rendered as colored segments in series.

***Unsupported DOT features***. The following features of Graphviz are not supported in the import:

1. HTML-like Labels
2. All node shapes are **not** supported **except** the following: triangle, diamond, ellipse, hexagon, octagon, parallelogram, rectangle, rect, box, and square. Msquare, Mcircle, and Mdiamond will be rendered the same as square, circle, and diamond respectively.
3. All arrow shapes are **not** supported **except** the following: vee, lvee, rvee, dot, normal, diamond, none, and tee. The arrow shapes odot, onormal, and odiamond will be rendered the same as dot, normal, and diamond respectively.
4. Edge curves. The value of the “pos” attribute is ignored when it is used as an attribute for edges, so dot-app will render edges only as straight lines between nodes.
5. The brewer colors chemes when using the “colorscheme” attribute.
6. The “tapered” and the “bold” edge style keywords
7. The “bold”, “diagonals”, “striped”, and “wedged” node style keywords
8. Default attribute values when grouping nodes with subgraphs. Only the default attribute values set within the root graph are considered.
9. Record-based Nodes. The label will not cause the node to become sectioned
10. Clusters
11. Edges that are rendered as colored parallel lines: These are made by assigning a color list without weights to the “color” attribute. Figure 10 depicts an example edge rendered in this manner.
12. Edges that are rendered as colored segments in series. These are made by assigning a color list with weights to the “color” attribute. Figure 11 depicts an example edge rendered in this manner.
13. Gradients applied to the network background

### Export

***Unsupported Cytoscape features***. When exporting the network as a GraphViz file, some Cytoscape information is lost because it cannot be represented in DOT format. dot-app does not keep a log of the information that is not transferred to the Graphviz file. The following information is lost:

**Visual information**

1. Custom graphics
  a. Images on nodes
  b. Charts on nodes
2. Edge bends
3. Nested network images contained in nodes
4. Arrowhead colors (they will appear the same color as the edge itself)
5. Certain line types
  a. Dash dot
  b. Contiguous arrow
  c. Backward slash
  d. Separate arrow
  e. Sinewave
  f. Vertical slash
  g. Zigzag
  h. Forward slash
  i. Parallel lines
6. Label positioning
  a. Edge labels only go on the midpoint of the edges
  b. Node label positions are selected at export
7. The V node shape
8. Target arrow shape
  a. Target arrow shape does not appear if set as a default (this was a bug in Cytoscape v3.2 that has been fixed since v3.3)
9. All annotations

**Non-visual information**

1. Node group information (groups that are collapsed are treated as a single node with no additional data)
2. All table data

## Use cases

Detailed below are two cases for dot-app. The first use case describes how a DOT file can be imported into Cytoscape. The second use case describes how a Cytoscape network can be exported as a DOT file. The DOT file used for import, the Cytoscape session file of the network, and the DOT file generated by dot-app during export are contained in ZIP 1 of the Supplementary Material.

### Import

**Figure 12.**
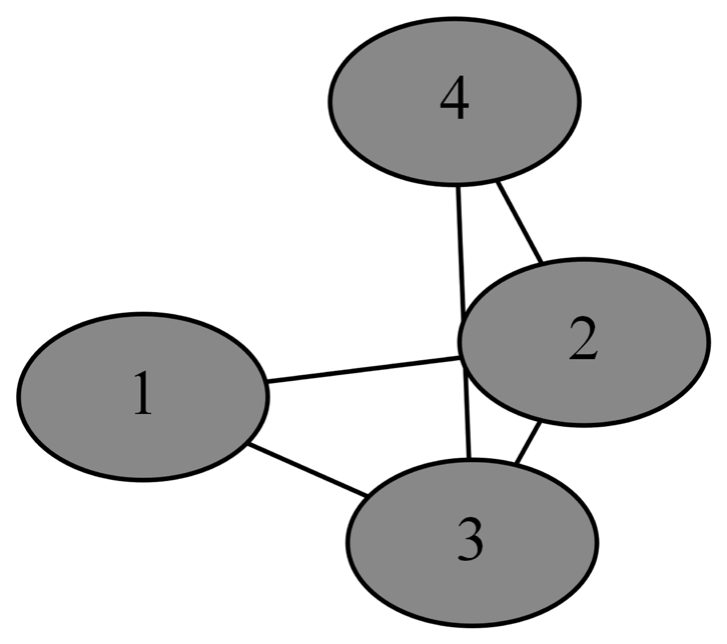
The PNG generated using Graphviz’s neato utility.

Our first use case details how we would use dot-app to view a Graphviz-created network in Cytoscape. We used Graphviz’s neato utility to create a DOT file with layout information and a PNG of the resulting network. The DOT file is shown below, and Figure 12 is the created PNG.



                        graph toy_example {
graph [bb="-85.648,-58.068,63.891,73.497",outputorder=edgesfirst, overlap=false];
node [fillcolor="#888888",label="\N",style=filled];
   1     [height=0.5,pos="-58.648,-8.4777",width=0.75];
   2     [height=0.5,pos="36.891,3.383",width=0.75];
   2 -- 1     [pos="10.278,0.079128 -2.8626,-1.5522 -18.68,-3.5159 -31.846,-5.1504"];
   3     [height=0.5,pos="12.665,-40.068",width=0.75];
   3 -- 1     [pos="-9.8989,-30.072 -18.223,-26.385 -27.653,-22.208 -35.986,-18.516"];
   3 -- 2     [pos="22.24,-22.895 23.933,-19.858 25.695,-16.698 27.386,-13.665"];
   4     [height=0.5,pos="8.8474,55.497",width=0.75];
   4 -- 2     [pos="18.03,38.433 21.097,32.734 24.516,26.38 27.592,20.664"];
   4 -- 3     [pos="9.5835,37.071 10.264,20.041 11.269,-5.1139 11.944,-22.022"];
}
                    


**Figure 13.**
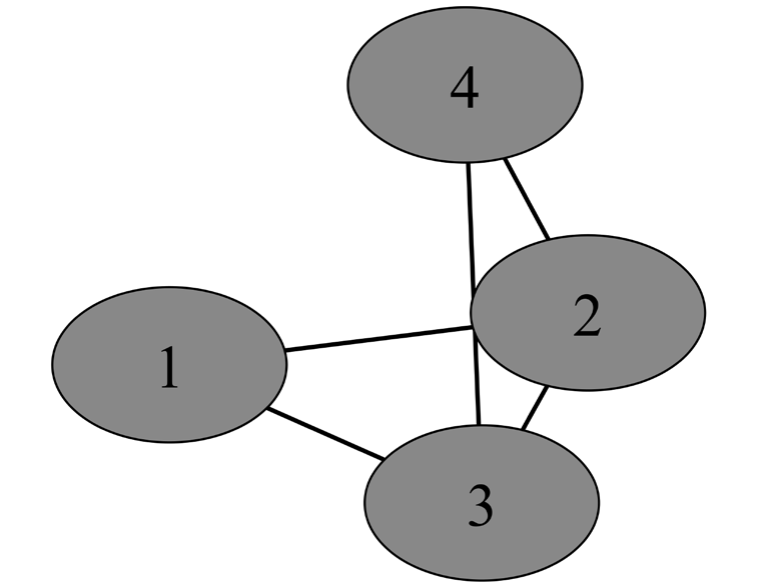
The DOT network as it looks in Cytoscape.

Figure 13 shows the result of the import into Cytoscape version 3.5.1. For a basic DOT file which uses attributes that have equivalents in Cytoscape, dot-app creates a faithful reproduction. A minor difference between the two is the width of the node borders. This is due to the difference between how Cytoscape and Graphviz render a node border with a width of 1.

### Export

**Figure 14.**
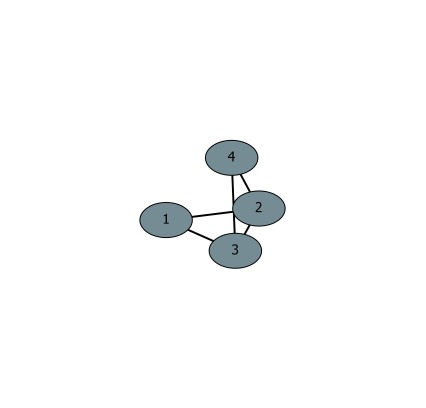
The PNG of the exported DOT file created with Graphviz’s neato utility.

In this second use case, we export the network in Figure 13 from Cytoscape. The output file is shown below. Figure 14 shows the PNG created by using Graphviz’s neato utility on the output file. With this DOT file, we are able to use Graphviz and other programs that accept DOT files, such as NetworkX and PyGraphviz.



                        graph toy_example {
bgcolor = "#FFFFFFFF"
splines = "false"
outputorder = "edgesfirst"
esep = "0"
pad = "2"
node [label = "\N",penwidth = "0.000000",height = "0.486111",width = "1.041667",tooltip = "",color = "#CCCCCCFF",fillcolor = "#888888FF",shape = "rectangle",style = "solid,rounded,filled",fontname = "SansSerif.plain",fontsize = "12",fontcolor = "#000000FF",fixedsize = "true",labelloc = "c"] 
edge [label = "",penwidth = "2.000000",tooltip = "",arrowhead = "none",arrowtail = "none",color = "#848484FF",fontname = "Dialog.plain",fontsize = "10",fontcolor = "#000000FF",style = "solid",dir = "both"]
"4§62" [label = "4",height = "0.500000",width = "0.750000",pos = "8.847400,55.497000"]
"3§63" [label = "3",height = "0.500000",width = "0.750000",pos = "12.665000,-40.068000"]
"2§64" [label = "2",height = "0.500000",width = "0.750000",pos = "36.891000,3.383000"]
"1§65" [label = "1",height = "0.500000",width = "0.750000",pos = "-58.648000,-8.477700"]
"4§62" -- "3§63" [color = "#404040FF"]
"4§62" -- "2§64" [color = "#404040FF"]
"3§63" -- "2§64" [color = "#404040FF"]
"3§63" -- "1§65" [color = "#404040FF"]
"2§64" -- "1§65" [color = "#404040FF"]
}
                    


In order to prevent labels from appearing cut off, dot-app adds a padding of 2 inches to its output file. The difference in the labels’ font is due to the font chosen in Cytoscape. In the output file shown above, we can see that the fontname attribute for the node default list is set to “Serif.plain”. This font is not an actual font family; rather, it is one of Java’s logical fonts ( https://docs.oracle.com/javase/tutorial/2d/text/fonts.html#logical-fonts). It is a font name that the Java Runtime Environment used and that maps to a physical font. When neato encounters the font name, it attempts to find an actual font named “Serif.plain”; if it cannot find one, it uses a default font.

## Testing

We verified the dot-app import and export functions separately. All of the files we used in testing are found in ZIP 2 of the Supplementary Material.

For import, we downloaded DOT files from Graphviz’s gallery page ( http://graphviz.org/Gallery.php) and wrote our own DOT files. We then ran Graphviz’s neato utility on these files to generate DOT files that contained layout information and PNG files to use as references. We then imported the DOT files to Cytoscape and visually compared the Graphviz-created PNG files to the Cytoscape display to validate the import process of dot-app.

For export, we loaded Cytoscape test session files ( https://github.com/cytoscape/cytoscape-tests/blob/master/docs/Session-Files/Session%20Files.md) into Cytoscape and exported individual networks from these sessions to DOT files, and then used Graphviz’s neato utility to create PNG files from these. We visually compared neato’s PNG output to the Cytoscape display to determine the correctness and completeness of the Cytoscape-to-DOT translation.

## Conclusion

This article describes the dot-app Cytoscape app, which enables a user to import a DOT file into Cytoscape and to export a Cytoscape network as a DOT file. We demonstrated the operation of dot-app and explained its implementation and the limitations of DOT-to-Cytoscape and Cytoscape-to-DOT translation. Finally, we explained typical use cases and how dot-app delivers value in each situation.

We recognize that as long as Cytoscape and Graphviz continue to add visual shapes and features, we will need to determine if these features are found in both applications and if they are we will need to update dot-app in order to provide support for these features. Dot-app’s primary goal was to allow Cytoscape to accept DOT files that represented common graph structures. We believe that dot-app as it is now can handle most of the more common DOT syntax people happen to use. However there are some, such as subgraphs, which people use that we were not able to support. We support anyone who finds dot-app lacking in some form and wishes to modify it.

## Software availability

Software available from:

http://apps.cytoscape.org/apps/dotapp

Latest source code:

https://github.com/idekerlab/dot-app

Archived source code as at the time of publication:

http://doi.org/10.5281/zenodo.821630 ^4^

License:

GNU General Public License v3

## Supplementary Material

**Supplementary File 1: List of DOT attributes that are ignored during import.**

**Supplementary File 2: Archive containing the files for the Use Cases section.** Contents are:

• neato_gen_example.gv: The DOT file generated with Graphviz’s neato utility

• example.cys: The Cytoscape session file that contains the example network

• cyto_gen_example.gv: The DOT file generated by dot-app for export

**Supplementary File 3: Archive containing the files for the Testing section.** All networks that were used for testing along with comparison images are stored in separate folders.
