# Supplementary material for: dot-app: a Graphviz-Cytoscape conversion plug-in
Source: F1000Res. 2017 Jul 10;5:2543. Originally published 2016 Oct 20. [Version 2] doi: 10.12688/f1000research.9751.2 (PMC5105883; doi:10.12688/f1000research.9751.2)
Supplement: Supplementary file 1 [file f1000research-5-13068-s0000.tgz › 1d34ecde-a0e1-477b-aa5f-45a86cbed311.pdf]

Supplementary File 1

Ignored DOT Attributes

This file contains all of the DOT attributes that are ignored when importing a DOT file into Cytoscape with dot-app. It is an edited version of the list of DOT attributes found on Graphviz's website (www.graphviz.org/content/attrs). The Used By column specifies which class of graph element can be modified by that attribute. G is for Graph, C is for Cluster, N is for Node, and E is for Edge. The attribute names are hyperlinks to their definition on the Graphviz site.

Name	Used By
Damping	G
K	GC
URL	ENGC
_background	G
area	NC
arrowsize	E
bb	G
center	G
charset	G
clusterrank	G
comment	ENG
compound	G
concentrate	G
constraint	E
decorate	E
defaultdist	G
dim	G
dimen	G
dir	E
diredgeconstraints	G
distortion	N
dpi	G
edgeURL	E
edgehref	E
edgetarget	E
edgetooltip	E
epsilon	G
esep	G
fixedsize	N
fontnames	G
fontpath	G
forcelabels	G
group	N
headURL	E
head_lp	E
headclip	E
headhref	E
headlabel	E
headport	E

**headtarget**	E
**headtooltip**	E
**href**	GCNE
**id**	GCNE
**image**	N
**imagepath**	G
**imagepos**	N
**imagescale**	N
**inputscale**	G
**labelURL**	E
**label_scheme**	G
**labelangle**	E
**labeldistance**	E
**labelfloat**	E
**labelhref**	E
**labeljust**	GC
**labelloc**	NGC
**labeltarget**	E
**labeltooltip**	E
**landscape**	G
**layer**	ENC
**layerlistsep**	G
**layers**	G
**layerselect**	G
**layersep**	G
**layout**	G
**len**	E
**levels**	G
**levelsgap**	G
**lhead**	E
**lheight**	GC
**lp**	EGC
**ltail**	E
**lwidth**	GC
**margin**	NCG
**maxiter**	G
**mclimit**	G
**mindist**	G
**minlen**	E
**mode**	G
**model**	G
**mosek**	G
**newrank**	G
**nodesep**	G
**nojustify**	GCNE
**normalize**	G
**notranslate**	G
**nslimit**<br>**nslimit1**	G
**ordering**	GN
**orientation**	N
**orientation**	G

**outputorder**	G
**overlap**	G
**overlap_scaling**	G
**overlap_shrink**	G
**pack**	G
**packmode**	G
**pad**	G
**page**	G
**pagedir**	G
**pencolor**	C
**peripheries**	NC
**pin**	N
**quadtree**	G
**quantum**	G
**rank**	S
**rankdir**	G
**ranksep**	G
**ratio**	G
**rects**	N
**regular**	N
**remincross**	G
**repulsiveforce**	G
**resolution**	G
**root**	GN
**rotate**	G
**rotation**	G
**samehead**	E
**sametail**	E
**samplepoints**	N
**scale**	G
**searchsize**	G
**sep**	G
**shapefile**	N
**showboxes**	ENG
**sides**	N
**size**	G
**skew**	N
**smoothing**	G
**sortv**	GCN
**splines**	G
**start**	G
**stylesheet**	G
**tailURL**	E
**tail_lp**	E
**tailclip**	E
**tailhref**	E
**taillabel**	E
**tailport**	E
**tailtarget**	E
**tailtooltip**	E
**target**	ENGC
**truecolor**	G

**vertices**	N
**viewport**	G
**voro_margin**	G
**xdotversion**	G
**xlp**	NE
**z**	N
