# Supplementary material for: dot-app: a Graphviz-Cytoscape conversion plug-in
Source: F1000Res. 2017 Jul 10;5:2543. Originally published 2016 Oct 20. [Version 2] doi: 10.12688/f1000research.9751.2 (PMC5105883; doi:10.12688/f1000research.9751.2)
Supplement: Peer review history

# Peer review: Referee response for version 2

**DOI:** 10.5256/f1000research.13068.r24109

**Published:** 2017-07-27

**Authors:** Matthias König

**Affiliations:** 1 Institute for Theoretical Biology, Humboldt University of Berlin, Berlin, Germany

We are satisfied that the authors have adequately addressed our comments in this new version.

 Minor points

 [1] Some of the test examples provided in Supplement 2 did not work with Cytoscape 3.5.1 and dot-app 0.9.4 and could not be imported

 dot-app_testing/Import/ER/ER.gv : Sorry! File did not comply to dot language syntax 

 dot-app_testing/Import/philo/philo.gv : Sorry! File did not comply to dot language syntax

 dot-app_testing/Import/unix/unix.gv : target node is not a member of this network

 All import examples distributed with the dot-app should work with dot-app (this should be part of the unittests). If the test examples do not work with the app there will be issues with real world examples. It is stated "We verified the dot-app import and export functions separately. All of the files we used in testing are found in ZIP 2 of the Supplementary Material." This seems not to be the case.

 Please fix the issue

 https://github.com/idekerlab/dot-app/issues/15

 [2] The app cannot be build from the provided repository after a clone.

 This is reported at

 https://github.com/idekerlab/dot-app/issues/14

 Please fix this issue. This is related to my initial review where I reported this issue.

I have read this submission. I believe that I have an appropriate level of expertise to confirm that it is of an acceptable scientific standard.

# Peer review: Referee response for version 2

**DOI:** 10.5256/f1000research.13068.r24107

**Published:** 2017-07-12

**Authors:** David J. Lynn, John Salamon

**Affiliations:** 1 European Molecular Biology Laboratory (EMBL) Australia Group, South Australian Health and Medical Research Institute, Adelaide, SA, Australia; 2 European Molecular Biology Laboratory (EMBL) Australia Group, South Australian Health and Medical Research Institute, Adelaide, SA, Australia

We are satisfied that the authors have adequately addressed our comments in this new version.

We have read this submission. We believe that we have an appropriate level of expertise to confirm that it is of an acceptable scientific standard.

# Peer review: Referee response for version 1

**DOI:** 10.5256/f1000research.10513.r17222

**Published:** 2016-12-20

**Authors:** Giovanni Scardoni

**Affiliations:** 1 Center for Biomedical Computing (CBMC), University of Verona, Verona, Italy

The article introduces an app to import/export Graphviz (.gv-.dot) files to/from Cytoscape. Since the Graphviz format is of wide usage, the app is very useful to the community.

 The article is well-written and the user-guide is complete and easy to read. 

 As minor comments I ask more details about which characteristics can be lost passing from a format to another. I tried some network choosing them randomly from internet, and sometimes I lost features that I didn't expect to lose.

 A table explaining which features are preserved and which not will be very useful in the paper. If there are problem of space maybe the implementation details can be described in supplementary materials and not in the main paper.

 The table should contains also short comments about the missing features that can be added in the future, and the problem that can be encountered implementing those characteristics that are not of immediate conversion.

 I think that with these updates the paper is suitable for indexing.

I have read this submission. I believe that I have an appropriate level of expertise to confirm that it is of an acceptable scientific standard.

# Response

**Published:** 2016-12-22

**Authors:** Ziran Zhang

**Affiliations:** University of California, San Diego, USA

Thank you Dr. Scardoni for your comment. We will take your valuable suggestions into consideration while we are refining the article. We appreciate your effort in examining the dot-app and the article for us in detail.

# Peer review: Referee response for version 1

**DOI:** 10.5256/f1000research.10513.r17125

**Published:** 2016-11-08

**Authors:** David J. Lynn, John Salamon

**Affiliations:** 1 European Molecular Biology Laboratory (EMBL) Australia Group, South Australian Health and Medical Research Institute, Adelaide, SA, Australia; 2 European Molecular Biology Laboratory (EMBL) Australia Group, South Australian Health and Medical Research Institute, Adelaide, SA, Australia

dot-app is a Cytoscape App that enables the inter-conversion of Graphviz and Cytoscape formatted files. As far as we are aware no similar application exists and this is therefore a potentially useful development.

 We found no problems with the usage of the app; it integrates well into Cytoscape, and can convert back and forth between formats without issue aside from the loss of information which is documented in the article. The code is readable and very well commented.

 We did have an issue when compiling from source, however. The DOT parsing jar from Alex Merz isn't being included properly when Maven builds the project.

 The major concern we had was that the concept for the application is really very straight-forward and following on from this, its functionality is therefore pretty limited. We wondered initially whether there was really a need for such an application, however, dot-app has been downloaded >1200 times so there is obviously a market for the tool. A remaining question, however, is whether the app justifies a 10-page paper? The paper itself reads a bit more like documentation and we would have preferred to have seen a more concise paper introducing the concepts together with more detailed documentation.

 We think that the paper could also benefit from a better discussion in the introduction of the motivation behind the development of dot-app. Can you give examples where one would want to create a network in one format and then convert it to another? How hard would it be to manually re-create the visualisation in Graphviz/Cytoscape?

 The application also appears very reliant on the parser developed by Alex Merz (this is not referenced properly). It would be good to clarify how much of the functionality is provided by this parser and how much is extended by dot-app.

 A key limitation of the app is that many of features of either format are lost when converting between them. This is because there isn’t an equivalent feature in the other format. It is good that the authors are clear about which ones cannot be converted but this problem still means that manual intervention would likely be needed to customise the desired style.

 One thing that could be improved is how the implicit default values are handled by dot-app. Couldn’t you set reasonable values for these if they are not specified in a particular visualisation to enable more faithful conversion of the visualisation style?

 One final concern is that it is very likely that the visual styles in both Graphviz and Cytoscape will continue to evolve over time. How will dot-app keep pace with these changes? Have the authors considered trying to get the Graphviz and Cytoscape communities to adopt at least the equivalent visual style features in both applications? Or at least to indicate in the Cytoscape and Graphviz documentation what is the equivalent feature (when there is one).

 Minor comment:

 Under Operation it would be good to start by explaining how to install the App (for novice Cytoscape users).

We have read this submission. We believe that we have an appropriate level of expertise to confirm that it is of an acceptable scientific standard, however we have significant reservations, as outlined above.

# Response

**Published:** 2016-11-08

**Authors:** Ziran Zhang

**Affiliations:** University of California, San Diego, USA

Thank you Dr. David Lynn and Dr. John Salamon for your comments. We completely agree with your suggestions, and we'll add those (e.g. network conversion examples, dot-app's future adaptation, detailed installation instructions, etc.) to our article. Please expect to see the changes you recommended in the second version of our article.

# Peer review: Referee response for version 1

**DOI:** 10.5256/f1000research.10513.r17148

**Published:** 2016-10-31

**Authors:** Matthias König

**Affiliations:** 1 Institute for Theoretical Biology, Humboldt University of Berlin, Berlin, Germany

The article "dot-app: a Graphviz-Cytoscape conversion plug-in" is well written and describes background, implementation and use of the application in a comprehensible way.

 ## major comments

 "The differences that arise between the Graphviz network and the Cytoscape network stem from how the two programs handle implicit default values. If a DOT attribute is omitted from the DOT file when using a Graphviz utility, an implicit default value for that attribute is used. The list of DOT attributes and their default values can be found on the Graphviz website (http://www.graphviz.org/content/attrs.html)."

 -> The implicit dot default values should be used as default values in dot-app for the Cytoscape styles. Users of the dot language rely on the default values and expect rendering tools to use them.

 -> Especially, in combination with dot-app writing the Cytoscape default values in the exported dot this results in unnecessary changes of the graph rendering in the round trip (dot -> cytoscape -> dot). This round trip should introduce minimal changes to the rendering.

 Dot default values should be used and the respective text passage and figure be updated.

 ## minor comments

 >>>

 In the implementation all visual styles are hard coded in the visual mapping via bipasses.

 It would be better to create node attributes for the dot attributes and subsequently use them in visual styles.

 In this way users have access to the dot attributes, can use them in other visual mappings, create derived node attributes from them, or use them in analyses.

 >>> It is unclear how dot height and width are transformed to Cytoscape node height and width. There is some scaling factor, but how this is actually handled should be described in the manuscript. What information is lost in the roundtrip due to scaling.

 For instance

 height = "0.486111",width = "1.041667"

 result in height > 40 in Cytoscape.

 >>> The dot node font color is not rendered correctly.

 See: https://github.com/idekerlab/dot-app/issues/7

 >>> Saving as *.gz always adds the .dot extension so that files are called *.gz.dot.

 If the user selects "name.gz" the file should be saved as 'name.gz', not as 'name.gz.dot'.

 >>>

 p5 "For the import function of dot-app, we used Java-based Parser for Graphviz Documents (JPGD), a Graphviz document parser made by Alexander Merz(5)"

 -> reference missing/wrong. There should be a reference for (5).

 >>>

 p5 "After JPGD creates the model objects, dot-app creates a corresponding "

 -> something wrong with first half-sentence, probably better: "After JPGD has created the model objects, ...

 >>>

 p6 "All other DOT attributes are ignored during the import process and have no effect on the visualization in Cytoscape."

 If the ignored DOT attributes can be listed, state them in the manuscript as list. This is very helpful to see what information should not be used in dot files if one wants full Cytoscape support of the file. If the unsupported attributes are only the features listed in "Unsupported DOT features" than this should be clearly stated.

 >>>

 p2 "The store contains nearly 300 apps that ..."

 At the state of review this is already >= 300. Add a date to the statement:

 -> The store contains nearly 300 apps (October 2016) that ...

 >>>

 Add reference for graphviz software/url

 see for instance http://www.graphviz.org/content/citing-graphviz-paper

 The preferred citation is

 @ARTICLE{Gansner00anopen,

     author = {Emden R. Gansner and Stephen C. North},

     title = {An open graph visualization system and its applications to software engineering},

     journal = {SOFTWARE - PRACTICE AND EXPERIENCE},

     year = {2000},

     volume = {30},

     number = {11},

     pages = {1203--1233}

 }

 and the URL is www.graphviz.org

 >>>

 p8 use cases

 Provide the *.gv files and Cytoscape files of the use cases in the supplement.

 This will provide the necessary materials to follow the provided examples. I tried to save the files from the pdf which did not work (due to line breaks in pdf), copying the examples from the HTML version of the manuscript worked. Adding the two example files to the supplementary information will improve this.

 >>>

 p9 testing

 Provide the url/repository for the test files and test results.

 There should be a page which shows all the comparison images with the respective dot files. In the article it is mentioned that this exists, but no resource is given for the test files and test results.

 >>>

 p9 conclusion

 "This article describes the dot-app Cytoscape app, which enables a user to import a DOT-formatted **here** into Cytoscape"

 -> word missing

 >>>

 p9 conclusion

 "we recognize that future versions of Cytoscape may introduce new visual effects"

 -> formulation. Better: 'visual shapes and styles", visual effects are something else.

 >>>

 p9 conclusion

 "we recognize that future versions of Cytoscape may introduce new visual effects (e.g., new arrow heads) that present opportunities for rendering DOT files more truly or for the loss of formatting information if the DOT format cannot represent them."

 -> second half of sentence does not make sense (... or for the loss of formating). Please reformulate to clarify what is meant.

 >>>

 The github release corresponding to the zenodo code is missing. The latest github release is 0.9.1 on 11.September, the zenodo was created in February. This makes it difficult to try to build the app from the mentioned source code, by checking out a corresponding tag.

 Which version is packed in zenodo? 0.9.2 ?

 Also the current app version in the app store is 0.9.3, but there is no 0.9.3 release on github.

 Please create a github release which is corresponding to the zenodo release and the mentioned code state of the publication.

 >>>

 I was unable to build the app from latest source code. The app is working via the app store, but in addition it should be possible to build the latest source code.

 https://github.com/idekerlab/dot-app/issues/8

 git clone https://github.com/idekerlab/dot-app.git

 mvn clean install

 results in

 [INFO] Compiling 16 source files to /home/mkoenig/git/dot-app/target/classes

 [INFO] ------------------------------------------------------------------------

 [INFO] BUILD FAILURE

 [INFO] ------------------------------------------------------------------------

 [INFO] Total time: 7.250 s

 [INFO] Finished at: 2016-10-31T12:48:57+01:00

 [INFO] Final Memory: 21M/238M

 [INFO] ------------------------------------------------------------------------

 [ERROR] Failed to execute goal org.apache.maven.plugins:maven-compiler-plugin:2.0.2:compile (default-compile) on project dot-app: Compilation failure: Compilation failure:

 [ERROR] /home/mkoenig/git/dot-app/src/main/java/org/cytoscape/intern/read/reader/NodeReader.java:[46,36] error: package com.alexmerz.graphviz.objects does not exist

 [ERROR]

 ...

 This should be fixed, I would recommend to setup some Continous Integration with travis to make sure the latest version always builds.

 see https://github.com/idekerlab/dot-app/issues/8

I have read this submission. I believe that I have an appropriate level of expertise to confirm that it is of an acceptable scientific standard, however I have significant reservations, as outlined above.

# Response

**Published:** 2016-10-31

**Authors:** Ziran Zhang

**Affiliations:** University of California, San Diego, USA

Thank you so much Dr. Matthias König for your insightful comments. We really appreciate your efforts of carefully testing the dot-app features and throughly examining our article. We thank you for bringing the problems you found to our attention. While waiting for another referee's upcoming peer review report, we will take a close look into the issues and address them for our next version.

# Peer review: Referee response for version 1

**DOI:** 10.5256/f1000research.10513.r17124

**Published:** 2016-10-27

**Authors:** Eric Bonnet

**Affiliations:** 1 Institute of Genomics, CEA (French Alternative Energies and Atomic Energy Commission), Evry, France

This article describes a new Cytoscape plugin called “dot-app” designed for the import and export of Graphviz files conforming to the DOT format for representing graph topologies, layouts and formatting.

 Graphviz is a popular set of open source software tools initiated at the AT&T Labs Research that can generate or process DOT files. Many other software tools are using Graphviz to represent all kinds of graphs.

 The DOT language support visual features and attributes that are not currently included by Cytoscape network formats. There is currently no Cytoscape plugin for the import and export of networks to the DOT language. Therefore the dot-app plugin may be useful to convert and import biological networks, and might be very interesting for the large community of Cytoscape users.

 The article is globally clear, well-organized and logically structured. I particularly appreciated the efforts of the authors to explain the details of the conversion process and exactly what kind of visual properties are imported and exported, including the properties that are not supported.

 I would suggest minor improvements to the article that could make it more precise and valuable for the readers.

1. Add a table for at least some of the main visual features and attributes supported by the DOT format and indicate which ones are supported by XGMML/GraphML. The sentence mentioning the differences in the introduction section is too vague.
2. Compile also in a small table some examples of applications (related to biology or not) that produces or consumes DOT files. Here also the paragraph discussing this in the introduction section is quite vague.

Minor comments:

- The reference to the JPGD parser do not appear in the bibliography.
- A few sentences on how to install the app in Cytoscape might be helpful.

I have read this submission. I believe that I have an appropriate level of expertise to confirm that it is of an acceptable scientific standard.

# Response

**Published:** 2016-10-30

**Authors:** Ziran Zhang

**Affiliations:** University of California, San Diego, USA

Thank you Dr. Bonnet for your valuable comments. We will look into the possible improvements and minor issues you brought up, and fix them in our next version. Again, we really appreciate your  detailed observations!
